# Role Captivity in Former Dementia Caregivers: Constrained Choice, Role Engulfment and Caregiver Wellbeing in Ageing Care

**DOI:** 10.3390/geriatrics11040094

**Published:** 2026-07-28

**Authors:** Barbara Plagg, Heidi Flarer, Petra Wlasak, Adolf Engl, Doris Hager von Strobele-Prainsack, Klaus Eisendle, Giuliano Piccoliori

**Affiliations:** 1 Institute of General Practice and Public Health, Claudiana–University College of Health Professions, 39100 Bolzano, Italy; 2 Central Hospital Bolzano/Bozen, South Tyrolean Health Service, 39100 Bolzano, Italy

**Keywords:** dementia caregiving, former caregivers, role captivity, constrained choice, role engulfment, family care, caregiver wellbeing, aging care

## Abstract

**Background/Objectives:** Family caregiving for people living with dementia is central to ageing and long-term care, but it is not only a practical arrangement but also a relational and structurally shaped experience. Care may be grounded in affection, responsibility, and connection, while still becoming difficult to refuse, share, or limit. Although caregiver burden is well documented, less is known about how former caregivers retrospectively appraise the caregiving role after active care has ended. This study examined role captivity among former family caregivers, focusing on constrained choice, subjective role engulfment, perceived support, and caregiver wellbeing. **Methods:** A retrospective cross-sectional mixed-methods survey was conducted among 180 former family caregivers of people living with dementia in Northern Italy. Quantitative analyses examined associations between role-captivity-related indicators and retrospective caregiving outcomes. Open-ended responses were analysed using theory-informed qualitative content analysis. **Results:** Constrained choice at caregiving uptake was reported by 22.2% of participants, and 56.1% met the criterion for subjective role engulfment. Overall, 60.0% would provide family care again, while only 28.3% would be willing to receive family care themselves. One in five reported caregiving-related health problems. In multivariable models, constrained choice was associated with lower willingness to provide care again (OR = 0.27, *p* = 0.002) and less positive retrospective appraisal (OR = 0.35, *p* = 0.036). Subjective role engulfment was independently associated with less positive appraisal (OR = 0.36, *p* = 0.008), while perceived support was associated with positive appraisal (OR = 6.31, *p* < 0.001) and perceived processing (OR = 2.68, *p* = 0.013). Qualitative findings showed that caregiving was often remembered as meaningful and relationally important, yet difficult to refuse, progressively all-consuming, and shaped by structural and informational barriers. **Conclusions:** Retrospective appraisal of family dementia caregiving depends not only on care demands, but also on whether care was experienced as chosen, bounded, shared, and supported. Role captivity helps identify when relationally meaningful care becomes difficult to sustain and highlights the need for caregiver-sensitive dementia care that protects agency, role boundaries, and access to timely support.

## 1. Introduction

Family caregiving for people living with dementia is a central component of ageing and long-term care. It enables many people with cognitive impairment to remain at home for longer, supports continuity and relational connection, and often compensates for limitations in formal care systems. At the same time, dementia caregiving is among the most demanding forms of unpaid family care: it is often prolonged, progressive, and marked by increasing dependency, behavioural and psychological symptoms, supervision needs, and substantial emotional and practical demands [1,2,3,4,5,6].

Existing research has extensively documented caregiver burden, psychological distress, social restriction, and health-related consequences among dementia caregivers. The caregiving stress process model has further shown how caregiving-related stress develops through primary stressors, secondary role strains, intrapsychic strains, coping resources, and social context [7]. However, burden-focused approaches do not fully distinguish between caregiving as demanding and caregiving as difficult to choose, share, limit, or step back from. This distinction is central to a role-captivity perspective. Research on informal caregiving has similarly shown that the assumption of care is often shaped by limited alternatives, family obligations, and relational expectations rather than representing a fully autonomous choice [8].

Role captivity and role engulfment are conceptually related to caregiver burden, but they specify different dimensions of the caregiving experience. Caregiver burden is commonly understood as a multidimensional response to caregiving demands, including physical, psychological, social, and financial strain. Burden-related research has repeatedly shown that care intensity, dependency, cognitive impairment, behavioural symptoms, time restrictions, role conflict, and insufficient support contribute to caregiver strain [3,9,10,11]. A role-captivity perspective does not deny these burdens; rather, it asks a more specific question: whether the caregiver experiences the role as one that can be chosen, shared, limited, and stepped back from.

Within the caregiving stress process tradition, role captivity has been treated as a subjective care-related stressor distinct from role overload: role overload captures the feeling that care demands exceed available resources, whereas role captivity captures the experience of being an involuntary incumbent of the caregiving role [7,12,13,14]. This distinction is empirically relevant. Longitudinal work on subjective care stressors has shown that role captivity and role overload may change differently over time and relate differently to personal, dyadic, family, and service-related resources; for example, family support and adult day service use were associated with lower captivity over time, while taking breaks from caregiving was more closely linked to lower overload [13]. Similarly, Liu et al. [14] showed that financial strain and employment changes were differentially associated with role captivity and overload among dementia family caregivers. Recent qualitative research has also examined how family caregivers of people with Alzheimer’s disease experience role captivity, further supporting its relevance as a distinct subjective dimension of dementia caregiving [15].

Role engulfment adds a further dimension by describing the displacement of other valued roles and aspects of the self as caregiving expands into everyday life. In Alzheimer’s caregiving, loss of self associated with caregiver-role engulfment has been linked to limited social contact and lack of other meaningful roles outside caregiving, supporting its distinction from care intensity alone [16]. Thus, high caregiving demands may contribute to burden, captivity, and engulfment, but they do not determine them in the same way. Similarly, intensive care situations may differ substantially in whether caregivers experience agency, role boundaries, shared responsibility, social participation, and preservation of self.

This perspective is particularly relevant in dementia care, where responsibilities often intensify gradually and may become concentrated on one family member. What begins as help with appointments, medication, finances, or household tasks may expand into constant availability, supervision, emotional labour, crisis management, and coordination with health and social care services. The consequences of caregiving may therefore depend not only on duration or intensity, but also on whether the role was experienced as freely entered, bounded, shared, and supported [14]. Informal care is also shaped by kinship, gendered expectations, proximity, employment status, perceived competence, service availability, and assumptions about who is responsible for sustaining care.

The present study forms part of the broader DEM-CARE South Tyrol project, which investigates both active and former family caregivers of people living with dementia. The former-caregiver component was introduced through an ethics-approved amendment during the ongoing study of active caregivers, after fieldwork highlighted a conceptual limitation of examining role captivity exclusively while caregiving is still ongoing. Active caregiving is often characterised by immediate care demands, continuous responsibility, progressive adaptation to increasing needs, and relational or normative obligations that may leave limited space for reflective distance. This does not imply that active caregivers are unable to validly evaluate their experiences; rather, it suggests that appraisal during ongoing caregiving captures a different vantage point from retrospective appraisal after the caregiving role has ended.

The distinction is particularly relevant to role captivity. Previous research has shown that the cessation of caregiving represents a major transition in which role-related strain, psychological distress, health consequences, and identity may persist, diminish, or be re-evaluated over time [12,17]. Studies of former dementia caregivers further demonstrate that the post-care period is a distinct phase of adjustment in which caregivers retrospectively make sense of the entire care trajectory, including its effects on health, identity, relationships, and future expectations [18,19]. Former caregivers therefore offer a distinct and complementary perspective rather than a substitute for active caregivers. Once active caregiving has ended, they can look back across the full trajectory—from entry into the role, through its gradual expansion and possible engulfment, to its eventual cessation—and assess whether care was experienced as chosen, bounded, shared, and supported.

Using a retrospective mixed-methods survey, this study examines role captivity among former dementia caregivers. It describes indicators of constrained choice and role engulfment, analyses their associations with willingness to provide family care again, perceived processing, positive retrospective appraisal, and self-reported caregiving-related health consequences, and explores how former caregivers describe constrained choice, role engulfment, support, and structural conditions in open-ended responses.

## 2. Materials and Methods

### 2.1. Study Design

This study used a retrospective cross-sectional mixed-methods survey design, combining quantitative analysis of structured survey data with qualitative content analysis of open-ended survey responses. The quantitative component examined the distribution of role-captivity-related indicators and their associations with retrospective caregiving appraisal among former dementia caregivers. The qualitative component was used to contextualise and deepen these findings by exploring how former caregivers described constrained choice, role engulfment, and structural conditions shaping their caregiving experience.

The study was conducted in South Tyrol, Northern Italy, as part of the DEM-CARE South Tyrol project. Data were collected through an anonymous online survey between September and December 2025.

### 2.2. Participants and Recruitment

The target population comprised adults who had previously provided unpaid home care to a person living with dementia and who were no longer actively caregiving for the person living with dementia to whom the survey referred. Eligibility criteria were: (1) age 18 years or older, (2) previous provision of unpaid home care to a person living with dementia within the last 10 years, and (3) no current active caregiving role for that person at the time of survey completion. No minimum interval since caregiving ended was specified. This was intentional, as the study aimed to capture variation across the post-care trajectory rather than restrict participation to a single predefined retrospective time point. Including caregivers whose care experience had ended recently as well as those with a longer post-care interval allowed short-, intermediate-, and longer-term perspectives on retrospective caregiving appraisal to be represented.

Participants were recruited through Alzheimer Südtirol Alto Adige (ASAA), general practitioners in the region, newsletters, mailing lists, social media, and associated dissemination channels. The study used a non-probabilistic convenience sample. The analytic sample comprised 180 former family caregivers of people living with dementia. Participants completed the survey in German or Italian according to their preferred language.

### 2.3. Ethics Approval, Consent, and Data Protection

The study was conducted in accordance with applicable ethical standards and data protection requirements. Ethical approval was obtained for the DEM-CARE South Tyrol project and its amendment for the retrospective survey of former caregivers. Participants provided informed consent electronically prior to survey entry. Data were collected anonymously, and no direct identifiers were recorded.

### 2.4. Survey Instrument and Measures

Data were collected using a structured online questionnaire designed to assess former caregivers’ retrospective accounts of dementia caregiving. The questionnaire combined closed-ended items with open-ended questions and covered sociodemographic characteristics, caregiving history and intensity, time since caregiving ended, support and service use, satisfaction with support, perceived voluntariness of caregiving uptake, retrospective appraisal, willingness to provide care again, willingness to receive family care oneself, and self-reported caregiving-related health consequences.

Constrained choice was assessed through the item on voluntariness of caregiving uptake; participants were classified as constrained if they indicated that they had not taken on caregiving voluntarily but had no real alternative. Subjective role engulfment was defined as endorsement of at least two of three caregiving-challenge indicators: continuous availability, lack of time for one’s own needs or leisure, and loneliness or lack of social support. Work-related restriction was examined as an additional indicator of role interference. Perceived support was assessed through overall satisfaction with support options and dichotomised as satisfied versus partly satisfied or dissatisfied. Caregiving intensity was based on reported daily caregiving hours near the end of the caregiving period.

For regression analyses, three dichotomous retrospective outcomes were examined: willingness to provide family care again, coded as yes versus no or unsure; positive retrospective appraisal, coded as yes versus mixed or no; and perceived processing of the caregiving experience, coded as processed well versus partly or not processed well. Self-reported caregiving-related health problems were examined descriptively and in bivariate analyses. Willingness to receive family care oneself was analysed descriptively and in cross-tabulation with willingness to provide care again. Open-ended responses were used to contextualise constrained choice, role engulfment, support, and retrospective appraisal.

### 2.5. Qualitative Analysis

Open-ended survey responses were analysed using theory-informed qualitative content analysis, combining deductive and inductive category development. The analysis included responses to seven open-ended survey items addressing participants’ entry into caregiving, experiences of support, advice to other caregivers, aspects they would approach differently, processing and appraisal of the caregiving experience, and additional comments.

The coding unit was the meaning-bearing passage, defined as a segment of text expressing one distinct experience, reason, need, appraisal, or recommendation. The complete response entry served as the context unit. Responses could contain several meaning-bearing passages and could therefore be assigned more than one code.

Analysis proceeded iteratively. First, all responses were read repeatedly to familiarise the researchers with the range and context of the material. Second, an initial deductive coding structure was developed from the study aims and conceptual framework. Third, this structure was refined inductively during coding by adding, differentiating, merging, or revising categories in response to the material. Fourth, category definitions, inclusion criteria, and coding boundaries were documented in an explicit coding framework. The final framework was then applied to the complete qualitative corpus. After coding was completed, category frequencies were calculated descriptively, and the qualitative findings were compared with the quantitative results to identify convergence, complementarity, and contextual explanation.

Initial coding was conducted by PW. BP reviewed the coding framework and ambiguous passages, which were discussed until consensus was reached. German and Italian responses were analysed in the original language, and illustrative quotations were translated into English for publication. Trustworthiness was supported through explicit category definitions and coding rules, review of the coding framework and ambiguous passages by a second researcher, consensus discussion, and retention of a response-level coding matrix as an audit trail.

Because responses to the different open-ended items were not linked at participant level, frequencies were calculated at the level of response entries rather than unique participants. A category was counted at most once within the same response entry, while individual entries could be assigned to more than one category. Frequencies were therefore treated as descriptive indicators of the empirical grounding of the categories rather than as prevalence estimates. The expanded coding framework is presented in Supplementary Table S3.

### 2.6. Statistical Analysis

Quantitative data were analysed descriptively and exploratorily. First, categorical variables were summarised using frequencies and percentages, with valid denominators reported where relevant. Descriptive analyses focused on caregiving context, role-captivity-related indicators, support, and retrospective outcomes.

Bivariate associations between role-captivity-related indicators and dichotomous outcomes were examined using cross-tabulations and chi-square tests or Fisher’s exact tests where expected cell counts were small. Effect sizes are reported as Phi coefficients. Multivariable associations were examined using binary logistic regression models. Models were built sequentially to distinguish the associations of role-captivity-related indicators from perceived support and caregiving intensity near the end of the caregiving period.

Given the exploratory nature of the study and the sample size, models were kept parsimonious. Covariates were selected based on conceptual relevance and empirical distribution rather than automated selection procedures. All quantitative analyses were conducted in IBM SPSS Statistics, version 22 (IBM Corp., Armonk, NY, USA). All tests were two-sided, with statistical significance set at α = 0.05.

## 3. Results

### 3.1. Sample Characteristics and Caregiving Context

The analytic sample comprised 180 former dementia caregivers. The sample was predominantly female (95.0%) and largely reflected intergenerational caregiving, with most respondents having cared for a parent (65.6%). Smaller groups reported having cared for a partner or spouse (13.3%), a parent-in-law (11.1%), or another relative (10.0%).

Caregiving trajectories were often prolonged. More than three-quarters of respondents had provided care for at least two years, including 43.9% who had cared for 2–5 years and 35.6% who had cared for more than five years. At survey completion, caregiving had ended less than one year previously for 25.6% of participants, 1–3 years previously for 34.4%, and more than three years previously for 40.0%.

Caregiving intensity increased substantially over time. Whereas daily caregiving hours at onset were more commonly in the lower range, intensity near the end of caregiving was markedly higher. Almost one-third of respondents (31.1%) reported providing more than 12 h of care per day near the end of the caregiving period, and a further 20.0% reported 6–12 h per day (Table 1). Caregiving ended following the death of the care recipient for 58.3% of participants (*n* = 105), transition to a residential care facility for 35.0% (*n* = 63), transfer of caregiving responsibility to another person for 5.6% (*n* = 10), and for other reasons for 1.1% (*n* = 2).

### 3.2. Role-Captivity-Related Indicators and Retrospective Outcomes

Indicators of constrained and role-engulfing caregiving were common (Table 2). Overall, 22.2% of participants (*n* = 40) reported constrained choice at caregiving uptake, indicating that they had taken on caregiving because they had no real alternative. Work-related restriction due to caregiving was reported by 36.1% (*n* = 65).

Subjective role engulfment was present in 56.1% of participants (*n* = 101). Only 12.8% (*n* = 23) endorsed none of the three role-engulfment indicators, whereas 23.9% (*n* = 43) endorsed all three. The broader profile of caregiving challenges showed that caregiving was frequently experienced as continuous responsibility: 78.0% (*n* = 138) reported being continuously “on call”, 62.7% (*n* = 111) reported psychological pressure such as fear or guilt, and 59.3% (*n* = 105) reported lack of time for their own needs or leisure. Further descriptive data on support, care-needs assessment, and preparedness are provided in Supplementary Table S1.

Retrospective outcomes suggested heterogeneous post-care appraisal. Overall, 60.0% (*n* = 108) indicated that they would provide family care again, whereas 40.0% (*n* = 72) would not or were unsure. Less than half (46.1%, *n* = 83) reported a clearly positive retrospective appraisal of caregiving, and 42.8% (*n* = 77) reported that they had processed the caregiving experience only partly or not well. Views on receiving family care oneself were divided: 28.3% (*n* = 51) would be willing to receive family care, 35.6% (*n* = 64) would not, and 36.1% (*n* = 65) were unsure. One in five respondents (20.0%, *n* = 36) reported health problems attributed to caregiving. Details on current self-rated health, recovery time after caregiving, and the types of caregiving-related health problems are shown in Supplementary Table S2.

### 3.3. Bivariate Associations Between Role-Captivity-Related Indicators and Retrospective Outcomes

Bivariate analyses showed that constrained choice and subjective role engulfment were more consistently associated with retrospective outcomes than work-related restriction (Table 3). Constrained choice was associated with a lower likelihood of positive retrospective appraisal (*p* = 0.007, Phi = 0.200) and, most strongly, lower willingness to provide care again (*p* < 0.001, Phi = 0.273). Its association with perceived processing was weaker (*p* = 0.076, Phi = 0.132), and no association was observed with caregiving-related health problems.

Subjective role engulfment was associated with poorer perceived processing (*p* = 0.018, Phi = 0.176), less positive retrospective appraisal (*p* = 0.001, Phi = 0.237), and lower willingness to provide care again (*p* = 0.020, Phi = 0.174). Its association with caregiving-related health problems was weaker and remained at trend level (*p* = 0.071, Phi = 0.134). Work-related restriction showed no meaningful bivariate associations with any of the examined outcomes and was therefore not retained in the multivariable models.

### 3.4. Multivariable Models of Retrospective Caregiving Appraisal

Logistic regression models were estimated for willingness to provide care again, positive retrospective appraisal, and perceived processing of the caregiving experience (Table 4). Models were built sequentially, first including constrained choice and subjective role engulfment, then adding perceived support, and finally adding care intensity near the end of caregiving.

Constrained choice emerged as the most robust correlate of willingness to provide care again. Across all models, participants who had taken on caregiving under conditions of constrained choice had substantially lower odds of reporting that they would provide care again. In the fully adjusted model, constrained choice remained significant (OR = 0.27, *p* = 0.002), while subjective role engulfment was attenuated and no longer reached conventional statistical significance (OR = 0.54, *p* = 0.088). Neither perceived support nor care intensity was independently associated with willingness to provide care again.

For positive retrospective appraisal, both constrained choice and subjective role engulfment remained relevant. In the fully adjusted model, constrained choice (OR = 0.35, *p* = 0.036) and subjective role engulfment (OR = 0.36, *p* = 0.008) were independently associated with lower odds of positive appraisal. Perceived support showed a strong positive association (OR = 6.31, *p* < 0.001). Care intensity near the end of caregiving was also associated with positive retrospective appraisal in the fully adjusted model (OR = 1.81, 95% CI 1.06–3.12, *p* = 0.031). Given the exploratory nature of the analysis, this association should be interpreted cautiously.

For perceived processing, the pattern differed. In the initial model, subjective role engulfment was associated with poorer perceived processing (OR = 0.49, *p* = 0.024), whereas constrained choice was not. Once perceived support was added, neither constrained choice nor subjective role engulfment remained significant. In the fully adjusted model, perceived support was the only significant predictor of reporting that the caregiving experience had been processed well (OR = 2.68, *p* = 0.013).

Overall, constrained choice was the most consistent correlate of reduced willingness to provide care again and less positive appraisal. Subjective role engulfment was particularly relevant for retrospective appraisal, while perceived support was strongly related to positive appraisal and emerged as the primary predictor for perceived processing.

### 3.5. Qualitative Findings

Across the seven open-ended survey items, 533 non-empty response entries were available. Of these, 516 contained material relevant to the analytic focus and were assigned at least one category, and 17 brief entries without codable content related to the study domains were retained in the audit record but not categorised. The analysis yielded four overarching themes and 17 subcategories: (1) constrained entry into caregiving, (2) expansion and engulfment of the caregiving role, (3) structural and informational amplifiers, and (4) retrospective appraisal and future limits. The complete category system, operational definitions, main source questions, and category frequencies are presented in Supplementary Table S3. Frequencies refer to coded response entries rather than unique participants and should not be interpreted as prevalence estimates.

Across the material, caregiving was rarely described as a simple voluntary decision followed by a stable and bounded set of tasks. Instead, participants portrayed a dynamic process in which relational commitment, constrained choice, progressive expansion of responsibility, and the availability or absence of support interacted over time. Positive and negative appraisals were not mutually exclusive: caregiving could be remembered as meaningful and relationally important while also being difficult to refuse, progressively all-consuming, or harmful to the caregiver’s own wellbeing.

### 3.6. Constrained Entry into Caregiving: Willingness Within Limited Alternatives

“It was self-evident that I would take it on.”

(P025)

Four subcategories described entry into caregiving: relational commitment, responsibility, love, or reciprocity (*n* = 10 coded response entries); no real alternative, sole availability, or unequal family participation (*n* = 18); gradual entry or role assignment through family position, gender, professional competence, or geographical proximity (*n* = 15); and the care recipient’s preference for home care or the lack of acceptable alternatives (*n* = 5).

These categories show why voluntariness was not adequately represented by a simple voluntary–involuntary dichotomy. Some participants explicitly wanted to care because of affection, loyalty, responsibility, or the wish to give something back. Yet willingness often coexisted with little practical room for refusal. Participants described being the only person available, the relative living closest, the family member regarded as professionally competent, or the person on whom responsibility implicitly fell because others did not participate. One participant described caregiving as “not necessarily voluntary, but simply because I was geographically the closest” (P055). Others described having gradually “slipped into” caregiving as occasional help developed into sustained responsibility. Constrained choice therefore emerged not as the absence of affection or commitment, but as restricted agency within relational, familial, and structural circumstances.

### 3.7. Expansion and Engulfment of the Caregiving Role: From Helping to Functioning

“There is simply no time to think about yourself; you just function when it is about family.”

(P019)

Three subcategories captured the progressive expansion of the caregiving role: escalating demands, continuous availability, and 24-h responsibility (*n* = 11); loss of personal time, privacy, social or family life, employment, or other valued roles (*n* = 23); and self-neglect, exhaustion, or health consequences (*n* = 38).

Participants described how caregiving expanded beyond its initial boundaries. What began as occasional assistance could develop into permanent availability, night-time responsibility, crisis management, organisational work, and constant anticipation of the next need. Role engulfment was therefore experienced not simply as providing many hours of care, but as the progressive displacement of other parts of life. Accounts referred to restricted social contacts, strain on partnerships and family life, employment consequences, and the disappearance of private or recovery time. Several participants retrospectively recognised that they had prioritised the person with dementia to such an extent that their own health and needs became secondary. As one participant stated, “I think I would take much better care of myself, but the support system simply does not allow for it” (P068). These accounts locate role engulfment at the intersection of escalating care needs, concentrated responsibility, and insufficient opportunities to step back.

### 3.8. Structural and Informational Amplifiers: When Support Comes Too Late or Not in Usable Form

“We waited almost a year for a reassessment—which took place on the day of her death.”

(P005)

Four subcategories described the structural and informational conditions surrounding care: delayed or inadequate services, bureaucracy, staffing shortages, and lack of places (*n* = 66); information, navigation, and preparedness gaps (*n* = 74); need for respite, practical help, shared responsibility, or formal care support (*n* = 201); and timely professional or family support as a protective buffer (*n* = 28). The high frequency of support-related entries should be interpreted in light of the survey items that explicitly asked about wished-for support and advice.

Participants reported delayed or inaccurate care-needs assessments, long waits for aids or services, lack of respite or residential places, financial barriers, and fragmented responsibilities between services. In these accounts, structural deficiencies did more than make caregiving inconvenient: they transferred unresolved care needs back to families and reduced caregivers’ ability to share or limit the role. Information gaps had a similar effect. Participants wished they had received earlier and clearer information about dementia progression, behavioural symptoms, legal and financial procedures, available services, and the point at which home care might no longer be sustainable. Several described entering caregiving as a “jump into cold water” (P079). Conversely, responsive professionals, competent home-care services, family sharing of responsibility, self-help groups, and practical guidance could widen caregivers’ room for manoeuvre. Support functioned not only by reducing tasks, but by preserving choice, recovery time, and boundaries around the caregiving role.

### 3.9. Retrospective Appraisal and Future Limits: Rejecting Unsupported Care, Not Care Itself

“I would do it again—but only with more help.”

(P027)

Retrospective accounts comprised six subcategories. Seeking help earlier, sharing responsibility, setting limits, and protecting oneself were the most frequently coded retrospective lessons (*n* = 153). Gratitude, closeness, meaning, learning, or personal growth appeared in 71 response entries, while persistent distress, guilt, regret, or incomplete processing appeared in 38. Seventeen entries explicitly combined positive meaning with substantial burden in the same account. Fifteen affirmed the past caregiving decision or indicated willingness to do the same again, whereas 12 rejected future caregiving or considered it acceptable only under better conditions.

Participants frequently resisted a simple positive–negative interpretation of caregiving. Some were deeply grateful for having accompanied a parent or partner and felt that they had done the right thing, while simultaneously describing exhaustion, lost time, damaged health, or unresolved distress. This coexistence of meaning and harm is important because it shows why role captivity should not be equated with lack of love or commitment. Retrospective advice repeatedly emphasised obtaining help before crisis points were reached, involving relatives more actively, preserving health and personal time, and accepting that one person could not sustainably meet all needs alone.

Taken together, the qualitative findings help explain the quantitative pattern. Constrained choice and subjective role engulfment mattered because participants distinguished between care that was demanding and care that became difficult to refuse, share, or limit. Structural and informational conditions shaped this distinction by narrowing or widening caregivers’ room for manoeuvre. Many participants did not reject caregiving itself; they rejected caregiving that was solitary, insufficiently supported, all-consuming, and structurally taken for granted.

## 4. Discussion

This mixed-methods survey examined role captivity among former family caregivers of people living with dementia and its association with retrospective caregiving appraisal. Three findings are central. First, constrained and role-engulfing caregiving experiences were common: about one-fifth of participants reported having taken on caregiving because they had no real alternative, and more than half met the study criterion for subjective role engulfment. Second, constrained choice was the most consistent correlate of less favourable retrospective appraisal, particularly lower willingness to provide family care again. Third, perceived support during caregiving was strongly associated with positive retrospective appraisal and perceived processing of the caregiving experience.

These findings suggest that former caregivers evaluate dementia caregiving not only in terms of care intensity or burden, but also in terms of agency, role boundaries, and support. This is important for ageing and long-term care because family caregiving is often treated as a stable resource within dementia care pathways. Our findings indicate that this resource may become fragile when care is experienced as difficult to refuse, difficult to share, and difficult to limit. Role captivity therefore adds specificity to burden-focused approaches by identifying a dimension of caregiving that is relational, structural, and highly relevant for caregiver wellbeing.

This distinction is also supported by the analytic pattern observed in the present study. Burden-related demands were clearly present: many participants reported continuous availability, lack of time for their own needs or leisure, high care intensity near the end of caregiving, and work-related restriction. However, these indicators did not all relate to retrospective outcomes in the same way. Work-related restriction showed no meaningful associations with perceived processing, positive retrospective appraisal, caregiving-related health problems, or willingness to provide care again. Care intensity near the end of caregiving also did not predict willingness to provide care again or perceived processing in the multivariable models. By contrast, constrained choice remained the most robust correlate of lower willingness to provide family care again, and subjective role engulfment remained independently associated with less positive retrospective appraisal. These findings suggest that the retrospective impact of caregiving cannot be understood from the amount of care provided alone. What also matters is whether intensive care was experienced as chosen or inescapable, bounded or all-consuming, and compatible or incompatible with preserving other roles and personal agency.

The qualitative findings help explain why constrained choice mattered. Caregiving uptake was rarely described as simple coercion. Rather, participants described care as emerging through affection, moral responsibility, geographic proximity, family expectations, professional background, or lack of alternatives. Care could therefore be meaningful and morally important while still leaving little room for refusal. Constrained choice should thus be understood not as a simple voluntary/involuntary binary, but as a continuum of constrained agency. This distinction is particularly relevant for relationship-centred dementia care: strong relational connection may motivate caregiving, but it does not necessarily protect caregivers from feeling trapped.

This interpretation also clarifies why constrained choice predicted lower willingness to provide care again. Reluctance to care again should not be read as rejection of the person cared for, nor as a rejection of family solidarity. Rather, many former caregivers appeared to reject a specific form of care: care that was insufficiently chosen, insufficiently supported, and difficult to limit. For geriatric and long-term care services, this is a critical point. If family care is to remain sustainable, willingness cannot simply be assumed; it must be supported through timely services, shared responsibility, and realistic alternatives.

Subjective role engulfment was also relevant, particularly for retrospective appraisal. This finding is consistent with earlier work on loss of self in Alzheimer’s caregiving, which described how engulfment in the caregiver role can displace identity, social roles, and everyday autonomy (Skaff & Pearlin, 1992) [16]. Participants in our study similarly described how caregiving expanded from helping into continuous responsibility, absorbing time, sleep, work, leisure, relationships, and emotional energy. Their retrospective advice—to seek help earlier, set limits, involve others, and protect one’s health—reflects the dimensions that had often been lost during caregiving. By contrast, work-related restriction showed no meaningful associations with retrospective outcomes. This does not make employment consequences unimportant, but suggests that later appraisal may depend more on the subjective experience of being unable to refuse, share, or limit care than on any single objective role restriction.

Perceived support emerged as a key contextual factor. It was strongly associated with positive retrospective appraisal and was the only significant predictor of perceived processing in the fully adjusted model. Qualitative accounts indicate why: support was most helpful when it was timely, practical, accessible, and able to translate complex needs into concrete help. Conversely, delayed assessments, fragmented services, financial barriers, lack of respite, and insufficient dementia-specific information narrowed caregivers’ room for manoeuvre and reinforced individual responsibility. Support should therefore be understood not only as task relief, but as a condition that preserves caregiver agency and may make the caregiving experience easier to process after care has ended.

The post-care perspective is an important contribution of this study. Former caregivers can reflect on whether the experience has been processed, whether they would provide care again, and whether they would accept family care for themselves. The discrepancy between willingness to provide family care again and reluctance to receive family care oneself warrants attention. It suggests that some former caregivers continue to value their own past commitment while simultaneously rejecting the idea of placing a similar burden on their own relatives. This finding points to a lasting imprint of intensive caregiving on future care preferences and expectations about family care. In ageing societies that rely heavily on informal care, such post-care attitudes should not be overlooked.

Several implications follow for geriatric and long-term care practice. Caregiver assessment should include perceived choice, continuous availability, lack of personal time, isolation, and the caregiver’s ability to step back from the role, rather than focusing only on care hours or task burden. Services should identify risk constellations early and offer support before crisis points are reached. Reassessment should occur as dementia progresses, because support that is sufficient at one stage may become inadequate later. Respite, dementia-specific guidance, service navigation, and shared care planning may help prevent family care from becoming all-consuming. Finally, former caregivers should not disappear from view once caregiving ends. If caregiving experiences continue to shape health, processing, and future care attitudes, post-care follow-up should be considered as part of caregiver-sensitive dementia care.

### Strengths and Limitations

This study has several strengths. It focuses on former dementia caregivers, a group less frequently studied than active caregivers, and thereby captures retrospective appraisal after the immediate demands of caregiving have ended. The mixed-methods design allowed quantitative associations to be interpreted alongside caregivers’ own accounts of how the role began, expanded, and became difficult to limit. The study also distinguishes role captivity from general caregiver burden and highlights perceived choice, role boundaries, and support quality.

Several limitations should be considered when interpreting the findings. The study used a non-probabilistic convenience sample, and the sample was predominantly female. This reflects the gendered distribution of dementia caregiving in many contexts, but limits the possibility of detailed gender comparisons. The retrospective cross-sectional design also precludes causal inference; participants’ current wellbeing and retrospective appraisal may have influenced recall. Role captivity was not measured with a dedicated validated scale, but operationalised through theoretically grounded indicators of constrained choice and role engulfment. Some variables were dichotomised for analysis, which increased interpretability but may have reduced nuance. The qualitative component was based on open-ended survey responses rather than in-depth interviews, limiting opportunities for probing and clarification. Finally, the sample was predominantly German-speaking, while Italian-speaking caregivers in South Tyrol were underrepresented. Findings should therefore be interpreted with attention to the study context and may not fully capture linguistic and cultural variation in caregiving experiences, family arrangements, and use of formal support services. Future longitudinal research should examine how changes in long-term care policy, service availability, employment protections, and financial support across the caregiving trajectory shape caregivers’ working lives, role captivity, role engulfment, and the sustainability of family care.

## 5. Conclusions

This study shows that retrospective appraisal among former family caregivers of people living with dementia is shaped by more than the level of care demands alone. Constrained choice, subjective role engulfment, and perceived support were central to how former caregivers evaluated the caregiving experience and whether they would consider providing family care again. These findings suggest that caregiver wellbeing in ageing care depends not only on reducing burden, but also on protecting agency, preserving role boundaries, and ensuring that family care is supported before it becomes all-consuming.

Role captivity offers a useful framework for understanding when relationally meaningful care becomes difficult to sustain. Family caregiving is often grounded in love, responsibility, and connection; however, these relational resources can become strained when care is silently assigned to one person, when support arrives too late, or when caregivers have limited room to refuse, share, or step back from the role. In this sense, role captivity highlights a critical threshold at which family care may remain morally meaningful but become practically and psychologically unsustainable.

For geriatric and long-term care systems, the implication is clear: caregiver-sensitive dementia care must move beyond recognising family caregivers as a resource and instead attend to the conditions under which care is provided. Assessment and support should include perceived choice, continuous availability, respite needs, service navigation, shared responsibility, and post-care follow-up. Addressing these dimensions is essential for advancing caregiving in ageing in ways that are relationally responsive, structurally supported, and attentive to the unequal conditions under which informal care is often sustained.

## Figures and Tables

**Table 1:** Sample characteristics (*n* = 180).

Characteristics of Caregiver	% (*n*)
Gender	
Female	95.0 (171)
Male	5 (9)
Other/prefer not to say	0 (0)
Language	
German	90.6 (163)
Italian	9.4 (17)
Education	
Compulsory school	7.2 (13)
Vocational/apprenticeship	35.6 (64)
High school	32.2 (58)
University/higher	25.0 (45)
Relationship with care recipient	
Parent	65.6 (118)
Partner/Spouse	13.3 (24)
Parent-in-law	11.1 (20)
Other	10.0 (18)
Care duration	
<1 year	4.4 (8)
1–2 years	16.1 (29)
2–5 years	43.9 (79)
>5 years	35.6 (64)
Time since end of caregiving	
<6 months	15.6 (28)
6 months–1 year	10.0 (18)
1–3 years	34.4 (62)
3–5 years	20.0 (36)
5–10 years	20.0 (36)
Reason caregiving ended	
Death of the care recipient	58.3 (105)
Transition to a residential care facility	35 (63)
Care assumed by another person	5.6 (10)
Other reason	1.1 (2)
Daily caregiving hours at onset	
1–2 h/day	33.3 (60)
2–3 h/day	24.4 (44)
3–6 h/day	17.8 (32)
6–12 h/day	8.3 (15)
>12 h/day	6.1 (11)
Other	10.0 (18)
Daily caregiving hours near the end	
1–2 h/day	4.4 (8)
2–3 h/day	11.1 (20)
3–6 h/day	25.0 (45)
6–12 h/day	20.0 (36)
>12 h/day	31.1 (56)
Other	8.3 (15)

**Table 2:** Role-captivity-related indicators and retrospective outcomes (*n* = 180).

Domain	Indicator/Category	*n* (%)
Role-captivity-related indicators	Constrained choice at caregiving uptake	40 (22.2)
	Work-related restriction due to caregiving	65 (36.1)
	Subjective role engulfment	101 (56.1)
Number of role-engulfment indicators endorsed	None	23 (12.8)
	One indicator	56 (31.1)
	Two indicators	58 (32.2)
	All three indicators	43 (23.9)
Retrospective outcomes	Would provide family care again: yes	108 (60.0)
	Positive retrospective appraisal: yes	83 (46.1)
	Processed caregiving experience well: yes	103 (57.2)
	Developed health problems due to caregiving: yes	36 (20.0)
Willingness to receive family care oneself	Yes	51 (28.3)
	No	64 (35.6)
	Unsure	65 (36.1)

Note. Values are presented as *n* (%). For dichotomous variables, only the affirmative or favourable category is shown. Subjective role engulfment was defined as endorsement of at least two of three indicators: continuous availability/being “on call”, lack of time for one’s own needs or leisure, and loneliness/lack of social support. Some role-captivity-related indicators are not mutually exclusive. Positive retrospective appraisal, perceived processing, and willingness to provide care again were dichotomised for regression analyses as shown.

**Table 3:** Bivariate associations between role-captivity-related indicators and retrospective caregiving outcomes.

Indicator	Outcome	Direction	*p*	Phi
Constrained choice	Processed caregiving experience well	Negative	0.076	0.132
	Positive retrospective appraisal	Negative	0.007	0.200
	Caregiving-related health problems	None	1.000	0.000
	Willingness to provide care again	Negative	<0.001	0.273
Work-related restriction	Processed caregiving experience well	None	0.708	0.028
	Positive retrospective appraisal	Negative	0.216	0.092
	Caregiving-related health problems	None	0.438	0.058
	Willingness to provide care again	None	0.751	0.024
Subjective role engulfment	Processed caregiving experience well	Negative	0.018	0.176
	Positive retrospective appraisal	Negative	0.001	0.237
	Caregiving-related health problems	Positive	0.071	0.134
	Willingness to provide care again	Negative	0.020	0.174

**Table 4:** Logistic regression models for retrospective caregiving outcomes.

Outcome/Model	Constrained Choice OR (95% CI), *p*	Subjective Role Engulfment OR (95% CI), *p*	Perceived Support OR (95% CI), *p*	Care Intensity Near End OR (95% CI), *p*	Nagelkerke’s R^2^
Panel A Willingness to provide care again					
M1	0.27 (0.13–0.57), <0.001	0.50 (0.26–0.94), 0.032	–	–	0.128
M2	0.27 (0.12–0.58), <0.001	0.49 (0.25–0.96), 0.039	1.37 (0.67–2.79), 0.389	–	0.145
M3	0.27 (0.12–0.62), 0.002	0.54 (0.27–1.10), 0.088	1.67 (0.77–3.62), 0.191	0.98 (0.60–1.60), 0.950	0.148
Panel B Positive retrospective appraisal					
M1	0.37 (0.17–0.81), 0.013	0.38 (0.21–0.71), 0.002	–	–	0.120
M2	0.34 (0.14–0.82), 0.016	0.47 (0.24–0.92), 0.029	3.85 (1.90–7.81), <0.001	–	0.234
M3	0.35 (0.13–0.93), 0.036	0.36 (0.17–0.76), 0.008	6.31 (2.76–14.40), <0.001	1.81 (1.06–3.12), 0.031	0.333
Panel C Perceived processing of the caregiving experience					
M1	0.55 (0.27–1.13), 0.105	0.49 (0.27–0.91), 0.024	–	–	0.061
M2	0.58 (0.28–1.24), 0.159	0.62 (0.33–1.19), 0.154	2.29 (1.13–4.62), 0.021	–	0.096
M3	0.52 (0.23–1.16), 0.109	0.60 (0.30–1.20), 0.146	2.68 (1.23–5.84), 0.013	1.00 (0.62–1.62), 0.996	0.124

Note. Values are odds ratios (ORs) with 95% confidence intervals (CIs) and *p* values. M1 included constrained choice and subjective role engulfment. M2 additionally included perceived support during caregiving. M3 additionally included care intensity near the end of caregiving. For all outcomes, higher odds indicate a greater likelihood of the favourable response category: willingness to provide care again, positive retrospective appraisal, or perceived processing of the caregiving experience.

## Data Availability

The data are not publicly available due to ethical and data protection restrictions. Aggregated information or further details may be made available from the corresponding author upon reasonable request and subject to applicable ethical and data protection requirements.

## Supplementary Materials

The following supporting information can be downloaded at: https://www.mdpi.com/article/10.3390/geriatrics11040094/s1, Table S1: Support, care-needs assessment, and preparedness during caregiving; Table S2: Health status, recovery, and caregiving-related health problems; Table S3: Expanded qualitative coding framework and category frequencies.

## References

[1] Brodaty H., Donkin M. (2009). Family caregivers of people with dementia. Dialogues Clin. Neurosci..

[2] Fonareva I., Oken B.S. (2014). Physiological and functional consequences of caregiving for relatives with dementia. Int. Psychogeriatr..

[3] Cheng S.-T. (2017). Dementia caregiver burden: A research update and critical analysis. Curr. Psychiatry Rep..

[4] Christian L.M., Wilson S.J., Madison A.A., Prakash R.S., Burd C.E., Rosko A.E., Kiecolt-Glaser J.K. (2023). Understanding the health effects of caregiving stress: New directions in molecular aging. Ageing Res. Rev..

[5] Deli D., Tsouvelas G., Roukas D., Mentis M. (2025). A systematic review of depressive and anxiety symptoms in caregivers of dementia patients. Psychiatriki.

[6] Schulz R., Sherwood P.R. (2008). Physical and mental health effects of family caregiving. Am. J. Nurs..

[7] Pearlin L.I., Mullan J.T., Semple S.J., Skaff M.M. (1990). Caregiving and the stress process: An overview of concepts and their measures. Gerontologist.

[8] Al-Janabi H., Carmichael F., Oyebode J. (2018). Informal care: Choice or constraint?. Scand. J. Caring Sci..

[9] de Almeida Mello J., Macq J., Van Durme T., Cès S., Spruytte N., Van Audenhove C., Declercq A. (2017). The determinants of informal caregivers’ burden in the care of frail older persons: A dynamic and role-related perspective. Aging Ment. Health.

[10] Lindt N., van Berkel J., Mulder B.C. (2020). Determinants of overburdening among informal carers: A systematic review. BMC Geriatr..

[11] Riffin C., Van Ness P.H., Wolff J.L., Fried T. (2019). Multifactorial examination of caregiver burden in a national sample of family and unpaid caregivers. J. Am. Geriatr. Soc..

[12] Aneshensel C.S., Pearlin L.I., Schuler R.H. (1993). Stress, role captivity, and the cessation of caregiving. J. Health Soc. Behav..

[13] Bangerter L.R., Liu Y., Kim K., Zarit S.H. (2019). Longitudinal trajectories of subjective care stressors: The role of personal, dyadic, and family resources. Aging Ment. Health.

[14] Liu Y., Dokos M., Fauth E.B., Lee Y.G., Zarit S.H. (2019). Financial strain, employment, and role captivity and overload over time among dementia family caregivers. Gerontologist.

[15] Zhang W., Xu W., Duan J., Tang J., Zhu Q., Pi X., Huang J. (2026). Exploring how family caregivers of people with Alzheimer’s disease experience role captivity: A qualitative study. Int. Psychogeriatr..

[16] Skaff M.M., Pearlin L.I. (1992). Caregiving: Role engulfment and the loss of self. Gerontologist.

[17] Robinson-Whelen S., Tada Y., MacCallum R.C., McGuire L., Kiecolt-Glaser J.K. (2001). Long-term caregiving: What happens when it ends?. J. Abnorm. Psychol..

[18] Corey K.L., McCurry M.K. (2018). When caregiving ends: The experiences of former family caregivers of people with dementia. Gerontologist.

[19] Jameson S., Parkinson L., Banbury A. (2020). After the care journey: Exploring the experiences of family carers of people living with dementia. Ageing Soc..

