# Supplementary material for: Role Captivity in Former Dementia Caregivers: Constrained Choice, Role Engulfment and Caregiver Wellbeing in Ageing Care
Source: Geriatrics (Basel). 2026 Jul 28;11(4):94. doi: 10.3390/geriatrics11040094 (PMC13512558; doi:10.3390/geriatrics11040094)
Supplement: Supplementary file 1 [file geriatrics-11-00094-s001.zip › geriatrics-4420692-supplementary.pdf]

Supplementary file

Supplementary Table S1. Support, care-needs assessment, and preparedness during caregiving.

Domain	Indicator	% (n)
**Support availability (N=180)**	No support received	3.9 (7)
	Support received from professional services	67.8 (122)
	Support received from family members	66.1 (119)
**Most helpful supports (valid N=173; multiple responses)**	Financial support (care allowance/benefits)	61.3 (106)
	Informal help from family/friends (e.g., household/shopping)	54.3 (94)
	Counselling by medical professionals	45.7 (79)
	Professional care/supervision services	34.1 (59)
	Psychological counselling/therapy	8.1 (14)
	Support groups	6.9 (12)
**Satisfaction with support options (valid N=173)**	Dissatisfied (very/rather)	28.3 (49)
	Partly satisfied/partly dissatisfied	37.6 (65)
	Satisfied (rather/very)	34.1 (59)
**Care-needs assessment (N=180)**	Formal assessment conducted	90.6 (163)
**Perceived assessment quality (valid N=173; multiple responses)**	Correct and timely	46.8 (81)
	Too late	35.3 (61)
	Inaccurate	23.7 (41)
**Preparedness/learning (N=180; multiple responses)**	Grew into the role over time	48.9 (88)
	Overwhelmed at the beginning due to lack of knowledge	39.4 (71)
	Knowledge gained is still useful today	31.1 (56)

Note. For multiple-response items, percentages represent the proportion of participants endorsing each option (“% of cases”); totals may exceed 100%. The table reports the most policy- and clinically relevant indicators.

Supplementary Table S2. Health status, recovery, and caregiving-related health problems.

Domain	Category / indicator	n (%)
**Current self-rated health (N = 180)**	Very good	30 (16.7)
	Good	101 (56.1)
	Fair	35 (19.4)
	Poor	13 (7.2)
	Very poor	1 (0.6)
**Recovery time after caregiving (N = 180)**	< 6 months	47 (26.1)
	6–12 months	55 (30.6)
	1–3 years	25 (13.9)
	> 3 years	6 (3.3)
	Still feel burdened	19 (10.6)
	Missing / not applicable	28 (15.6)
**Caregiving-related health problems (N = 180)**	Developed health problems due to caregiving: yes	36 (20.0)
	Developed health problems due to caregiving: no	144 (80.0)
**Type of health problems among those reporting caregiving-related health problems (n = 36)**	Sleep disturbances	21 (58.3)
	Burnout / chronic exhaustion	14 (38.9)
	Depressive symptoms	13 (36.1)
	Chronic back pain	10 (27.8)
	Headaches / migraine	10 (27.8)
	Anxiety disorders / panic attacks	8 (22.2)
	Cardiovascular problems	6 (16.7)
	Digestive problems	6 (16.7)
	Worsening of pre-existing conditions	4 (11.1)
	Other	12 (33.3)

Note. Values are presented as n (%). For the type of caregiving-related health problems, percentages refer to the subgroup reporting health problems due to caregiving (n = 36). Multiple responses were possible; therefore, percentages in this section do not sum to 100%.

Supplementary Table S3. Expanded qualitative coding framework and category frequencies.

Main theme	Subcategory	Operational definition / inclusion rule	Main source questions	n
**Constrained entry into caregiving**	Relational commitment, responsibility, love, and reciprocity	Caregiving uptake was framed as an expression of affection, loyalty, moral responsibility, gratitude, or a wish to give something back.	Voluntariness / entry into caregiving; final comments	10
	No real alternative, sole availability, or unequal family participation	Caregiving was taken on because no realistic alternative was perceived, nobody else was available, or responsibility was concentrated unequally on one person.	Voluntariness / entry into caregiving; final comments	18
	Gradual entry or role assignment through family, gender, profession, or proximity	Care responsibilities accumulated incrementally or were implicitly assigned because of family position, gendered expectations, professional competence, a shared household, or geographical proximity.	Voluntariness / entry into caregiving; final comments	15
	Care-recipient preference for home care or lack of acceptable alternatives	Home caregiving was shaped by the care recipient's wish to remain at home, rejection of institutional care, or the absence, inaccessibility, or unaffordability of acceptable formal alternatives.	Voluntariness / entry into caregiving; final comments	5
**Expansion and engulfment of the caregiving role**	Escalating demands, continuous availability, and 24-hour responsibility	Care expanded into permanent availability, night-time duties, vigilance, crisis management, or near-continuous responsibility.	Advice; what would be done differently; processing, appraisal, and final comments	11
	Loss of own time, privacy, social or family life, employment, or other valued roles	Caregiving displaced leisure, privacy, relationships, employment, family time, or other valued roles and aspects of the caregiver's life.	Advice; what would be done differently; processing, appraisal, and final comments	23
	Self-neglect, exhaustion, and health consequences	Responses described psychological or physical exhaustion, neglect of personal needs, loss of recovery time, or caregiving-related health consequences.	Advice; what would be done differently; processing, appraisal, and final comments	38
**Structural and informational amplifiers**	Delayed or inadequate services, bureaucracy, staffing shortages, and lack of places	Structural barriers included delayed or inaccurate assessment, bureaucracy, waiting lists, staffing shortages, fragmented services, financial barriers, and lack of respite, day-care, residential, or equipment provision.	Wished-for support; advice; what would be done differently; final comments	66

Main theme	Subcategory	Operational definition / inclusion rule	Main source questions	n
	Information, navigation, and preparedness gaps	Caregivers lacked timely and understandable information about dementia, disease progression, behavioural symptoms, entitlements, procedures, available services, or where to obtain help.	Wished-for support; advice; what would be done differently; final comments	74
	Need for respite, practical help, shared responsibility, or formal care support	Responses called for earlier or greater respite, home help, day or residential care, practical assistance, shared family responsibility, or other formal support.	Wished-for support; advice; what would be done differently; final comments	201
	Timely professional or family support as a protective buffer	Existing support was described as timely, competent, accessible, and able to reduce isolation, share responsibility, or preserve the caregiver's room for manoeuvre.	Wished-for support; advice; what would be done differently; final comments	28
**Retrospective appraisal and future limits**	Meaning and burden coexisting in the same retrospective account	The same response explicitly combined meaning, gratitude, closeness, or having done the right thing with exhaustion, restriction, loss, or harm.	Processing and appraisal comments; advice; what would be done differently; final comments	17
	Seeking help earlier, sharing responsibility, setting limits, and protecting oneself	Retrospective advice emphasised obtaining or accepting help earlier, delegating, involving others, setting boundaries, preserving personal time, or protecting health.	Processing and appraisal comments; advice; what would be done differently; final comments	153
	Persistent distress, guilt, regret, or incomplete processing	Responses described continuing guilt, grief, anger, regret, psychological distress, health effects, or a sense that the caregiving experience had not been fully processed.	Processing and appraisal comments; advice; what would be done differently; final comments	38
	Gratitude, closeness, meaning, learning, or personal growth	Caregiving was associated with relational closeness, gratitude, meaning, learning, pride, personal growth, or the sense of having done the right thing.	Processing and appraisal comments; advice; what would be done differently; final comments	71
	Affirmation of past caregiving or willingness to do the same again	Participants affirmed their past caregiving decision or stated that they would provide the same care again.	Processing and appraisal comments; advice; what would be done differently; final comments	15

Main theme	Subcategory	Operational definition / inclusion rule	Main source questions	n
	Refusal of future caregiving or willingness only under better conditions	Participants rejected future caregiving or considered it acceptable only with substantially greater support, shared responsibility, clearer limits, or a shorter and more manageable care trajectory.	Processing and appraisal comments; advice; what would be done differently; final comments	12

Note. n represents the number of coded response entries in which the category was identified. Because responses to the seven open-ended items were not linked at participant level, frequencies do not represent unique participants. Each category was counted at most once per response entry; entries could receive multiple categories, so frequencies do not sum to the 516 coded response entries. Frequencies are descriptive and should be interpreted in light of the number and wording of the source questions.
